# Development and validation of a deep learning model to predict survival of patients with esophageal cancer

**DOI:** 10.3389/fonc.2022.971190

**Published:** 2022-08-10

**Authors:** Chen Huang, Yongmei Dai, Qianshun Chen, Hongchao Chen, Yuanfeng Lin, Jingyu Wu, Xunyu Xu, Xiao Chen

**Affiliations:** ^1^ Shengli Clinical College of Fujian Medical University, Department of Thoracic Surgery, Fujian Provincial Hospital, Fuzhou, China; ^2^ Shengli Clinical College of Fujian Medical University, Department of Oncology, Fujian Provincial Hospital, Fuzhou, China; ^3^ College of Mathematics and Data Science (Software College), Minjiang University, Fuzhou, China

**Keywords:** deep learning, esophageal cancer, DeepSurv, survival prediction, treatment recommendation

## Abstract

**Objective:**

To compare the performance of a deep learning survival network with the tumor, node, and metastasis (TNM) staging system in survival prediction and test the reliability of individual treatment recommendations provided by the network.

**Methods:**

In this population-based cohort study, we developed and validated a deep learning survival model using consecutive cases of newly diagnosed stage I to IV esophageal cancer between January 2004 and December 2015 in a Surveillance, Epidemiology, and End Results (SEER) database. The model was externally validated in an independent cohort from Fujian Provincial Hospital. The C statistic was used to compare the performance of the deep learning survival model and TNM staging system. Two other deep learning risk prediction models were trained for treatment recommendations. A Kaplan–Meier survival curve was used to compare survival between the population that followed the recommended therapy and those who did not.

**Results:**

A total of 9069 patients were included in this study. The deep learning network showed more promising results in predicting esophageal cancer-specific survival than the TNM stage in the internal test dataset (C-index=0.753 vs. 0.638) and external validation dataset (C-index=0.687 vs. 0.643). The population who received the recommended treatments had superior survival compared to those who did not, based on the internal test dataset (hazard ratio, 0.753; 95% CI, 0.556-0.987; P=0.042) and the external validation dataset (hazard ratio, 0.633; 95% CI, 0.459-0.834; P=0.0003).

**Conclusion:**

Deep learning neural networks have potential advantages over traditional linear models in prognostic assessment and treatment recommendations. This novel analytical approach may provide reliable information on individual survival and treatment recommendations for patients with esophageal cancer.

## Introduction

Esophageal cancer (EC) is the most common gastrointestinal tumor globally and ranked seventh in terms of incidence and sixth in terms of overall mortality in 2018 (1). Despite progress in the treatment and management of EC in recent years, the long-term survival of patients undergoing esophagectomy remains poor (17.1-55%) (2). The benefits of adjuvant therapy have been debated and inconclusive. Therefore, it is very important to stratify the risk and make individualized treatment recommendations for newly diagnosed patients.

The American Joint Committee on Cancer (AJCC) tumor, node, and metastasis (TNM) staging system is widely used to stratify disease risk, predict patient survival outcomes, and make decisions regarding cancer treatment (3). However, the AJCC staging system is not accurate enough to predict the survival of patients with esophageal cancer who received multimodality treatment (4). Moreover, survival rates in stage-matched cohorts varies widely (5). To improve the precision of EC survival estimations, nomograms have gained popularity as a method for predicting outcomes (6–9). A nomogram is a Cox proportional hazard (CPH) model designed to allow straightforward graphical calculation of the probability of a specific outcome, such as esophageal cancer-specific survival (ECSS). However, these models have some limitations in time–event prediction for the clinical management of patients with cancer, including an accurate assessment of overall survival (OS) and progression time (10). Moreover, it is not sufficient to consider only the linear relationship of clinical features when making treatment decisions (11). Therefore, a better model that focuses on nonlinear variables is required.

Deep learning networks provide insights into the highly complex linear/nonlinear associations between prognostic clinical features and the individual risk of death (12). Matsuo et al. developed a deep learning neural network model that exhibited superior performance compared to the CPH model for survival prediction in women with cervical cancer (13). Katzman et al. developed a CPH deep neural network called DeepSurv (14), which can be used to predict the effects of patient covariates on patient survival. The authors demonstrated that DeepSurv performed better than other state-of-the-art survival methods for modeling the interactions between a patient’s covariates and treatment effectiveness. She et al. found that DeepSurv has a potential benefit in prognostic evaluation and treatment recommendations with respect to lung cancer-specific survival (15).

In this study, we first explored the performance of the DeepSurv model in analyzing the real-world clinical data of patients with esophageal cancer. Second, we evaluated the reliability of the DeepSurv model in providing treatment recommendations based on individual characteristics.

## Materials and methods

### Eligibility criteria and clinical information

For the training cohort, we selected patients from the Surveillance, Epidemiology, and End Results (SEER) database: SEER 18 Regs Custom Data [with additional treatment fields], Nov 2018 Sub [1975-2016 varying]. We obtained permission to access the database by signing the SEER Research Data Agreement form and submitting it *via* email. The inclusion criteria were as follows: (1) pathologically confirmed primary stage I to IV EC (only adenocarcinoma and squamous cell carcinoma) between January 2004 and December 2015 and (2) the presence of one malignant primary lesion. The exclusion criteria were as follows: (1) missing clinicopathological data and (2) patients with perioperative mortality (mortality within 30 days after operation). Baseline patient information (sex, age, race, and marital status), tumor characteristics (primary site, histologic grade, histologic type, and TNM stage), SEER code (CS extension, CS mets at DX, regional nodes examined, regional nodes positive, and CS tumor size), and treatment details were collected (**Table S1**). The outcome of interest in this study was ECSS, which was defined as the interval from diagnosis to death as a result of EC. These cases were randomly divided into training and test cohorts at a ratio of 8 to 2. Another test cohort from our database was provided to externally validate the DeepSurv model. After obtaining institutional review board approval from Fujian Provincial Hospital, we selected patients received esophagectomy from January 2011 to December 2016 in Fujian Provincial Hospital (CHINA dataset), and these patients were completely distinct from those in the SEER database. The requirement for informed consent was waived owing to the retrospective nature of this study. The inclusion criteria specified patients with pathologic stage I to IV; and complete resection of microscopic tumors. Patients with secondary malignancies, perioperative mortality, and missing of clinical records were excluded from the study. A flow chart of dataset construction is shown in **Figure S1**.

### Deep learning model design

We performed survival analysis based on the deep learning model DeepSurv described by Katzman el al. (14) to predict individual patient outcomes. DeepSurv is a multilayer fully connected network composed of input, hidden, and output layers. Nonlinear features are introduced through the hidden layer of the neural network to fit the proportional hazard function under nonlinear conditions. The expression for the hidden layer is *f*(*X*)=*Relu*(*θX*+*b*), where Relu is a nonlinear activation function, θ is the parameter matrix, X is the input feature, and b is the bias term. The deep learning model learns the complex relationship between individual covariates and treatment effects by replacing the linear combination of features *h_β_* (*x*) = *β^T^x* with the output *h_θ_* (*x*) of the nonlinear network layer. This model simulates the actual clinical treatment risk of the population and has a strong generalization ability. The model uses weight decay regularization, batch normalization, and dropout to prevent overfitting. The loss function of deep learning is set as the Cox partial likelihood with constraints and is defined as


$$l\left(\theta \right)=-\frac{1}{N_{E}}\sum_{i,E_{i}=1}\left({\hat{h}}_{\theta }\left(x\right)-log\sum_{j\in R\left(T_{i}\right)}e^{{\hat{h}}_{\theta }\left(x\right)}\right)+\alpha {∥\theta ∥}_{2}^{2}$$

where *θ* is a parameter of the neural network, α is the regular coefficient, and ${\hat{h}}_{\text{θ}}\left(x\right)$ is the output of the model. N_E_ represents the number of patients who eventually died (14). The loss function is used to estimate the degree of inconsistency between the predicted value and the real value of the model. The smaller the loss function is, the better the robustness of the model is. The model uses the Adam optimization algorithm to optimize the loss function and update the parameters (16).

Random search was used to optimize the hyper-parameters of the network because it is more efficient than grid search when dealing with high-dimensional data (17). Random search finds optimal model hyper-parameters by selecting a random combination of parameters from the search space. In this study, we performed a randomized hyper-parameter search over the number of layers in [2, 7], the number of neurons in each layer in [4, 100], the learning rate in [0.00001, 0.01]. The model structure was optimized using 500 iterations of random search on the training set for predicting the survival of patients with EC. Hyper-parameters search showed increasing the number of hidden layers can lead to improved model performance until the number of hidden layers exceeded three. So a 5-layer network with three hidden layers was a good choice. Similarly, the number of neurons in each layer was optimized according to random search results.

### Data analysis

**Figure 1:**
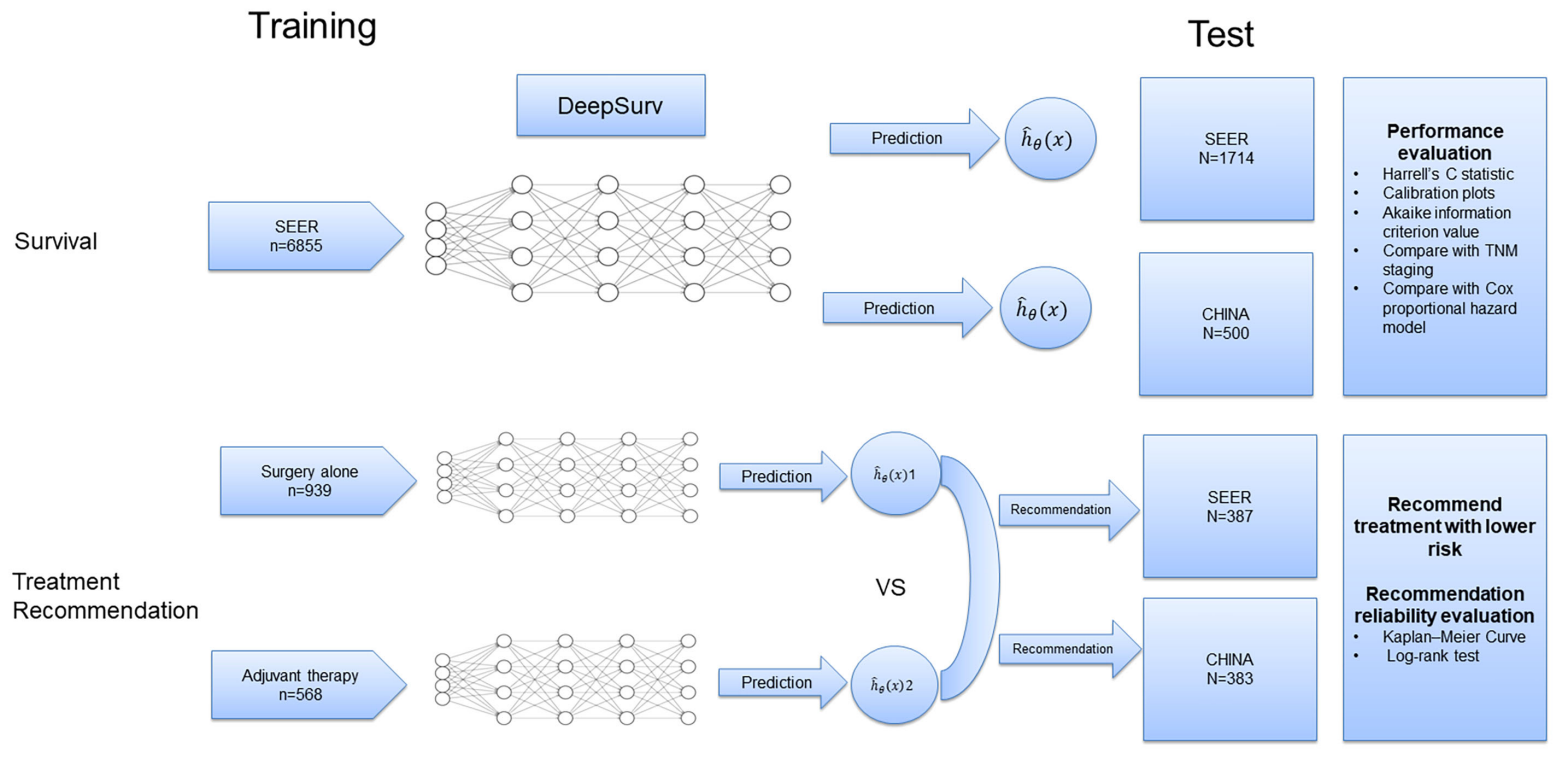
Diagram of the study procedure.

First, a 5-layer neural network was trained to predict the ECSS of the patients in the training dataset (n=6855) (**Figure 1**). To validate the prediction performance, we used Harrell’s C statistic and calibration plots to evaluate network discrimination and calibration in the SEER (n=1714) and CHINA (n=500) test datasets. An additional CPH regression model was performed following the backward stepwise approach, using all variables included in the DeepSurv model. The CPH model is a classic model for clinical survival analysis that uses a linear function *h_β_* (*x*) = *β^T^x* to estimate the true risk function *h*(*x*). The prediction performances of the DeepSurv, CPH, and TNM staging models were compared using the C statistic.

Next, patients who underwent esophagectomy were screened from the three datasets and divided into the surgery alone and adjuvant therapy groups according to whether they received adjuvant radiotherapy and/or chemotherapy (**Figure S1**). The data in the surgery alone (n=939) and adjuvant therapy (n=568) training sets were used to train two DeepSurv risk prediction models. The survival risk for each patient on different treatment regimens in the test set (SEER: n=387; CHINA: n=383) was predicted, and treatment options with a lower risk were recommended (**Figure 1**). Finally, patients in the test set were divided into two groups based on whether the recommended treatment was used. We used the Kaplan–Meier method to analyze the ECSS between different groups, and the log-rank test was used to compare survival curves.

### Statistical Analysis

A two-sided P value less than 0.05 was considered statistically significant. The Akaike information criterion (AIC) value was calculated to assess the risk of overfitting (18). The deep learning model was developed using Python (version 3.6.7). The CPH regression model and the C statistical were determined by survival, survminer, and rms packages with R statistical software (Version 4.2.0), and the survival curves were plotted using GraphPad Prism 7 (GraphPad Software).

## Results

### Baseline characteristics and survival

According to the inclusion criteria, 9069 patients with EC were included in this study. The baseline clinical characteristics of the patients are shown in **Table 1**. A total of 8,569 patients from the SEER database were included. The median (interquartile range) age was 65 (23-101), and the major race was white (7293[95.1%]). The majority of tumors were in the lower third of the esophagus (6091[71.1%]), stage IV disease (1753[20.4%]), and adenocarcinoma (5883[68.6%]). The median (interquartile range) follow-up time was 15 (0-155) months. During the follow-up time, 5469 patients (63.8%) died with their cause of death attributed to EC. There were 500 patients diagnosed with EC in the CHINA database. In that dataset, 227 patients died with their cause of death attributed to EC over a median (interquartile range) follow-up time of 47 (2-155) months.

**Table 1:** Characteristics of patients in the whole data sets of survival analysis.

Characteristic	Data set, No. (%)					
	Training		Test1 (SEER)		Test2 (CHINA)	
Age at diagnosis, median (range), y	65	(23,101)	65	(23,97)	62	(34,88)
Race						
White	5854	(85.4)	1439	(84.0)	0	(0)
Black	648	(9.5)	176	(10.2)	0	(0)
Others^1^	353	(5.1)	99	(5.8)	500	(100)
Sex						
Male	5505	(80.3)	1386	(80.9)	350	(70.0)
Female	1350	(19.7)	328	(19.1)	150	(30.0)
Marital status						
Widowed	673	(9.8)	206	(12.0)	18	(3.6)
Married	4150	(60.5)	1008	(58.8)	404	(80.8)
Single	1168	(17.0)	290	(16.9)	47	(9.4)
Divorced	780	(11.5)	182	(10.6)	28	(5.6)
Separated	70	(1.0)	25	(1.5)	3	(0.6)
Unmarried or domestic partner	14	(0.2)	3	(0.2)	0	(0)
Primary site						
Upper third of esophagus	367	(5.4)	76	(4.4)	53	(10.6)
Middle third of esophagus	1133	(16.5)	307	(17.8)	251	(50.2)
Lower third of esophagus	4883	(71.2)	1208	(70.5)	169	(33.8)
Overlapping lesion of esophagus	301	(4.4)	78	(4.6)	8	(1.6)
Cervical esophagus	115	(1.7)	25	(1.5)	1	(0.2)
Abdominal esophagus	56	(0.8)	20	(1.2)	18	(3.6)
Histologic type						
Adenocarcinoma	4690	(68.4)	1193	(69.6)	14	(2.8)
Squamous cell carcinoma	2165	(31.6)	521	(30.4)	486	(97.2)
Grade						
Grade III, Poorly differentiated	3277	(47.8)	803	(46.8)	120	(24.0)
Grade II, Moderately differentiated	3110	(45.4)	807	(47.1)	347	(69.4)
Grade I, Well differentiated	468	(6.8)	104	(6.1)	33	(6.6)
Stage (AJCC 7th)						`
IA	657	(9.6)	168	(9.7)	25	(5.0)
IB	869	(12.7)	228	(13.3)	70	(14.0)
IIA	321	(4.7)	63	(3.7)	60	(12.0)
IIB	1316	(19.2)	327	(19.1)	155	(31.0)
IIIA	1342	(19.6)	332	(19.4)	111	(22.2)
IIIB	454	(6.6)	123	(7.2)	50	(10.0)
IIIC	496	(7.2)	120	(7.0)	28	(5.6)
IV	1400	(20.4)	353	(20.6)	1	(0.2)
T stage						
T1a	558	(8.1)	130	(7.5)	27	(5.4)
T1b	506	(7.4)	124	(7.2)	64	(12.8)
T1 NOS	1026	(15.0)	284	(16.6)	0	(0)
T2	962	(14.0)	210	(12.3)	95	(19.0)
T3	2908	(42.4)	743	(43.3)	302	(60.4)
T4a	307	(4.5)	70	(4.1)	4	(0.8)
T4b	246	(3.6)	61	(3.6)	8	(1.6)
T4 NOS	342	(5.0)	92	(5.4)	0	(0)
N stage						
N0	3373	(49.2)	859	(50.1)	279	(55.8)
N1	2361	(34.4)	554	(32.3)	140	(28.0)
N2	788	(11.5)	223	(13.0)	63	(12.6)
N3	333	(4.9)	78	(4.6)	18	(3.6)
M stage						
M0	5455	(79.6)	1361	(79.4)	499	(99.8)
M1	1400	(20.4)	353	(20.6)	1	(0.2)
Therapy to primary site						
None	3553	(51.8)	925	(54.0)	0	(0)
Esophagectomy	3095	(45.1)	729	(42.5)	500	(100)
Local tumor destruction	207	(3.1)	60	(3.5)	0	(0)
Radiation sequence						
No radiation	4724	(68.9)	1243	(72.5)	314	(62.8)
Radiation prior to surgery	1529	(22.3)	331	(19.3)	69	(13.8)
Radiation after surgery	513	(7.5)	120	(7.0)	117	(23.4)
Others^2^	89	(1.3)	20	(1.2)	0	(0)
Chemotherapy						
Yes	2177	(31.8)	1143	(66.7)	282	(56.4)
No	4678	(68.2)	571	(33.3)	218	(43.6)
ECSS						
Alive	2486	(36.3)	614	(35.8)	273	(54.6)
Dead	4369	(63.7)	1100	(64.2)	227	(45.4)

^1^American Indian/AK Native, Asian/Pacific Islander.

^2^Radiation before and after surgery/Surgery both before and after radiation/Sequence unknown, but both were given/Intraoperative radiation with other radiation before/after surgery. ECSS, Esophageal Cancer-Specific Survival; AJCC, American Joint Committee on Cancer; NOS, Not otherwise specific.

### Training curves

**Figure 2:**
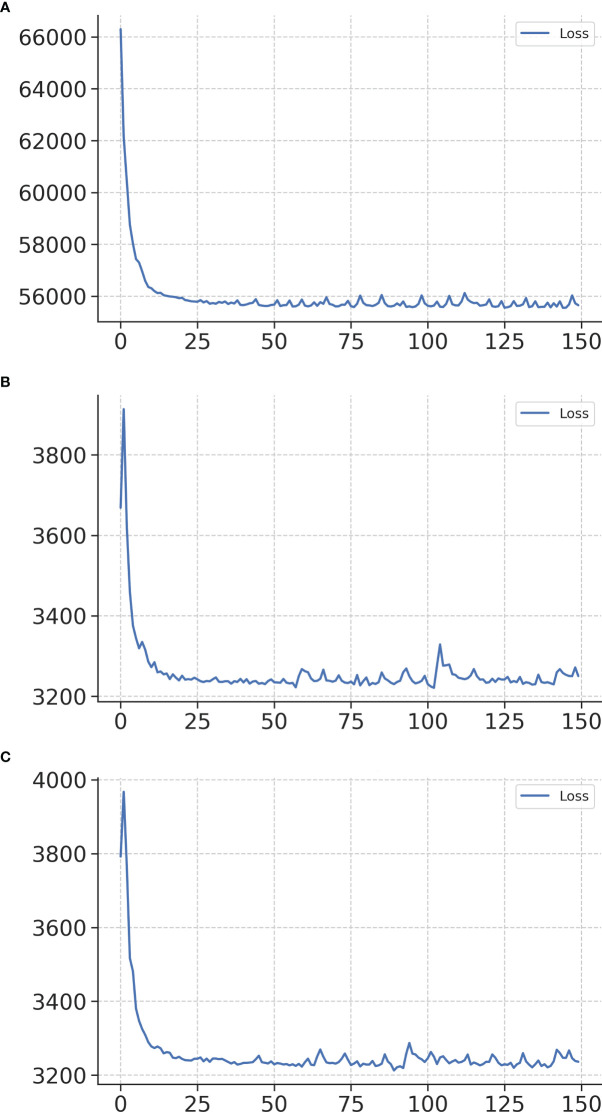
Training loss curves of networks in the survival network **(A)**, treatment recommendation network of surgery alone **(B)**, and treatment recommendation network of adjuvant therapy **(C)**. The x-axis represents the number of iterations, and the y-axis represents the loss function.

**Figure 2A** shows the training loss curves of the survival network. The accuracy of the model during the training process was represented by the loss function. The loss function continues to decrease with an increase in the number of iterations. Within 200 epochs, the curve is relatively smooth, indicating that the model has a strong fitting ability and can quickly learn effective discriminant feature information from the training samples. While the model has a good fitting ability on the training set, it also maintains a good generalization ability on the test and validation sets. **Figures 2B**, **C** show the training loss curves for the two treatment recommendation models. Owing to the decrease in data diversity in the recommended training set, the decrease in the loss function was smoother than that of the survival model.

### Calibration and validation of the prognostic model for ECSS

**Figure 3:**
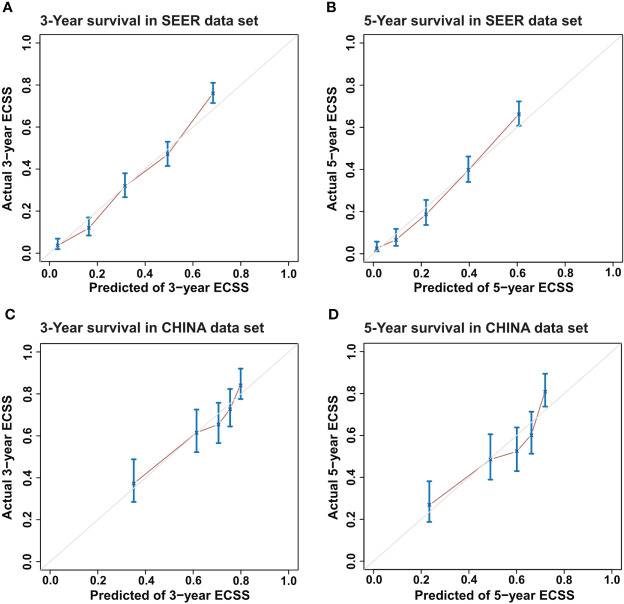
Calibration plots for Esophageal Cancer-Specific Survival (ECSS) for the DeepSurv model. **(A)** 3-year and **(B)** 5-year ECSS of Surveillance, Epidemiology, and End Results (SEER) dataset and **(C)** 3-year and **(D)** 5-year ECSS of CHINA dataset.

First, a calibration plot was used to test the consistency between the 3-year and 5-year ECSS predicted by the DeepSurv model and the actual survival of each case in the test dataset. The calibration plot (**Figure 3**) shows that most points are arranged around a straight line at an angle of 45° to the x-axis, indicating that the predicted value is very close to the actual value.

In the SEER test set, the prediction performance of DeepSurv was better than that of TNM staging (C-index=0.753 vs. 0.638), and similar results were obtained using the CHINA test set (C-index=0.687 vs. 0.643). The C-index of the surgery alone model in the SEER and CHINA test sets was 0.734 and 0.689, respectively. The C-index of the adjuvant therapy model in the SEER and CHINA test sets was 0.721 and 0.634, respectively. The feature component weightings in the DeepSurv model are listed in **Table S2**.

The performances of the CPH and DeepSurv models in predicting the ECSS were also compared. **Table 2** lists the factors included in the CPH model. The DeepSurv model performed better than the CPH model in the SEER test set (C-index=0.753 vs. 0.728) and CHINA test set (C-index=0.687 vs. 0.655). The AIC values of the TNM stage, CPH, and DeepSurv model were 70521, 69331 and 69262, respectively.

**Table 2:** The variables included in the Cox proportional hazard model.

Variable	Univariable Analysis	Multivariable Analysis			
	*P*	HR	95% CI	β	*P*
Age	<0.001	1.0119	1.0090-1.0147	0.0118	<0.001
Sex	0.77	–	–	–	–
Race	<0.001	1.0714	1.0099-1.1367	0.0689	0.022
Marital status	<0.001	1.0453	1.0234-1.0676	0.0443	<0.001
Primary site	0.043	1.0706	1.0297-1.1131	0.0682	<0.001
Histologic	<0.001	0.8748	0.8117-0.9428	-0.1338	<0.001
Grade	<0.001	1.1706	1.1116-1.2328	0.1576	<0.001
Therapy to primary site	<0.001	0.5273	0.4779-0.5818	-0.64	<0.001
Sequence of radiation	<0.001	0.983	0.9499-1.0173	-0.0171	0.328
Chemotherapy	0.28	–	–	–	–
Regional nodes examined	<0.001	0.9813	0.9764-0.9862	-0.0189	<0.001
Regional nodes positive	<0.001	1.0865	1.0720-1.1013	0.0835	<0.001
Stage (AJCC 7^th^)	<0.001	1.156	1.1170-1.1964	0.145	<0.001
T stage	<0.001	0.936	0.8974-0.9763	-0.0661	0.002
N stage	<0.001	0.9604	0.9130-1.0103	-0.0404	0.118
M stage	<0.001	0.7126	0.5885-0.8628	-0.3389	<0.001
CS tumor size	<0.001	1.0023	1.0016-1.0029	0.0023	<0.001
CS extension^1^	<0.001	1.0011	1.0007-1.0015	0.0011	<0.001
CS mets at DX^2^	<0.001	1.0156	1.0118-1.0194	0.0155	<0.001

^1^Details are available from: https://web2.facs.org/cstage0205/esophagus/Esophagus_bbb.html.

^2^Details are available from: https://web2.facs.org/cstage0205/esophagus/Esophagus_hbg.html.

HR, Hazard Ratio; CI, Confidence Interval; AJCC, American Joint Committee on Cancer; NOS, Not otherwise specific.

### Treatment recommender

**Figure 4:**
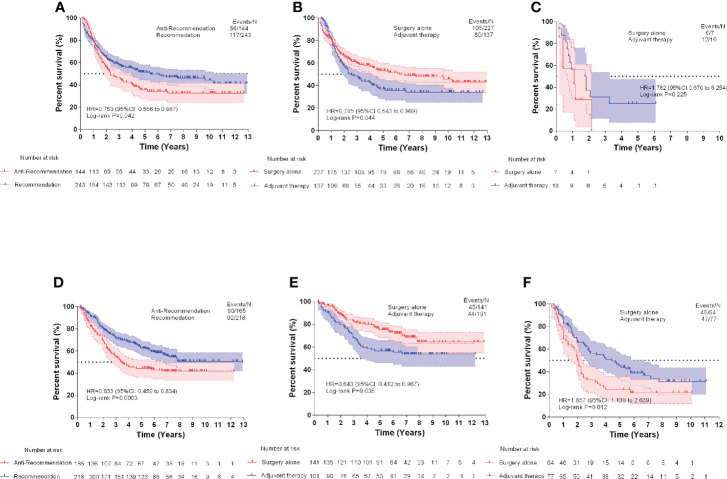
Esophageal cancer-specific survival comparisons of Surveillance, Epidemiology, and End Results (SEER) test dataset **(A)**, SEER surgery alone recommendation test dataset **(B)**, and SEER adjuvant therapy recommendation test dataset **(C)**. Esophageal cancer-specific survival comparisons of CHINA test dataset **(D)**, CHINA surgery alone recommendation test dataset **(E)**, and CHINA adjuvant therapy recommendation test dataset **(F)**.

The baseline clinical characteristics of the patients included in the treatment recommendation study are presented in **Table 3**. By plotting the Kaplan–Meier survival curve, the clinical prognosis of the two groups of patients (those who followed the treatment recommendation vs. those who did not follow the treatment recommendation) were compared (**Figure 4**). The survival rate of patients who followed the treatment recommendations was significantly higher than that of patients who did not (SEER: hazard ratio [HR], 0.753; 95% CI, 0.556-0.987; P=0.042 vs. CHINA: HR, 0.633; 95% CI, 0.459-0.834; P=0.0003). ECSS favored surgery alone compared with surgery combined with adjuvant therapy in the subgroup with surgery alone recommendation (SEER: HR, 0.745; 95% CI, 0.543-0.983; P=0.044 vs. CHINA: HR, 0.643; 95% CI, 0.412-0.967; P=0.035). In the subgroup with adjuvant therapy recommendation, patients who only received surgical treatment experienced significantly worse ECSS than those who received surgery combined with adjuvant therapy in the CHINA dataset (HR, 1.657; 95% CI, 1.138-2.639; P=0.012). No significant difference in ECSS was observed between the two treatment opinions in the SEER dataset (HR, 1.782; 95% CI, 0.670-6.252; P=0.225).

**Table 3:** Characteristics of patients in the whole data set of treatment recommendation.

Characteristic	Data set, No. (%)								
	Training			Test1 (SEER)			Test2 (CHINA)		
Age at diagnosis,median(range),y	64	(26,92)		63	(29,90)		60	(34,88)	
Race									
White	1340	(88.9)		334	(86.3)		0	(0)	
Black	85	(5.7)		27	(7.0)		0	(0)	
Others^1^	82	(5.4)		26	(6.7)		383	(100)	
Sex									
Male	1252	(83.1)		324	(83.7)		269	(70.2)	
Female	255	(16.9)		63	(16.3)		114	(29.8)	
Marital status									
Widowed	115	(7.6)		27	(7.0)		10	(2.6)	
Married	990	(65.7)		248	(64.1)		335	(87.5)	
Single	234	(15.5)		68	(17.6)		23	(6)	
Divorced	153	(10.2)		39	(10.1)		14	(3.7)	
Separated	14	(0.9)		4	(1.0)		1	(0.3)	
Unmarried or domestic partner	1	(0.1)		1	(0.3)		0	(0)	
Primary site									
Upper third of esophagus	32	(2.1)		12	(3.1)		47	(12.3)	
Middle third of esophagus	200	(13.3)		49	(12.7)		194	(50.7)	
Lower third of esophagus	1195	(79.2)		302	(78.0)		117	(30.5)	
Overlapping lesion of esophagus	51	(3.4)		12	(3.1)		17	(4.4)	
Cervical esophagus	10	(0.7)		2	(0.5)		1	(0.3)	
Abdominal esophagus	19	(1.3)		10	(2.6)		7	(1.8)	
Histologic type									
Adenocarcinoma	1182	(78.4)		304	(78.6)		14	(3.7)	
Squamous cell carcinoma	325	(21.6)		83	(21.4)		369	(96.3)	
Grade									
Grade III, Poorly differentiated	647	(42.9)		168	(43.4)		84	(21.9)	
Grade II, Moderately differentiated	700	(46.4)		180	(46.5)		278	(72.6)	
Grade I, Well differentiated	160	(10.7)		39	(10.1)		21	(5.5)	
Regional nodes examined, mean ± standard deviation	15.10 ±11.40			13.99±11.20			25.10 ±14.10		
Regional nodes positive, mean ± standard deviation	1.67±3.49			1.81±3.67			1.36±2.65		
Stage (AJCC 7^th^)									
IA	329		(21.8)	76		(19.6)	23		(6)
IB	252		(16.7)	60		(15.5)	61		(15.9)
IIA	83		(5.5)	22		(5.7)	37		(9.7)
IIB	310		(20.6)	75		(19.4)	119		(31.1)
IIIA	220		(14.6)	55		(14.2)	75		(19.6)
IIIB	119		(7.9)	38		(9.8)	46		(12)
IIIC	119		(7.9)	33		(8.5)	21		(5.5)
IV	75		(5.0)	28		(7.2)	1		(0.3)
T stage									
T1a	214		(14.2)	52		(13.4)	23		(6)
T1b	350		(23.2)	79		(20.4)	57		(14.9)
T1 NOS	35		(2.3)	10		(2.6)	0		(0)
T2	251		(16.7)	54		(14.0)	66		(17.2)
T3	592		(39.3)	165		(42.6)	229		(59.8)
T4a	9		(0.6)	5		(1.3)	2		(0.5)
T4b	6		(0.4)	3		(0.8)	6		(1.6)
T4 NOS	50		(3.3)	19		(4.9)	0		(0)
N stage									
N0	843		(55.9)	218		(56.3)	219		(57.2)
N1	354		(23.5)	83		(21.4)	94		(24.5)
N2	192		(12.8)	55		(14.2)	55		(14.4)
N3	118		(7.8)	31		(8.0)	15		(3.9)
M stage									
M0	1432		(95)	359		(92.8)	382		(99.7)
M1	75		(5.0)	28		(7.2)	1		(0.3)
Radiation sequence									
No radiation	1173		(77.8)	312		(80.6)	314		(82)
Radiation after surgery	334		(22.2)	75		(19.4)	69		(18)
Chemotherapy									
Yes	522		(34.6)	145		(37.5)	167		(43.6)
No	985		(65.4)	242		(62.5)	216		(56.4)
Actual treatment									
Surgery alone	939		(62.3)	234		(60.5)	205		(53.5)
Adjuvant therapy	568		(37.7)	153		(39.5)	178		(46.5)
ECSS									
Alive	765		(50.8)	184		(47.5)	201		(52.5)
Dead	742		(49.2)	203		(52.5)	182		(47.5)

^1^ American Indian/AK Native, Asian/Pacific Islander.

ECSS, Esophageal Cancer-Specific Survival; AJCC, American Joint Committee on Cancer; NOS, Not otherwise specific.

### Model visualization

**Figure 5:**
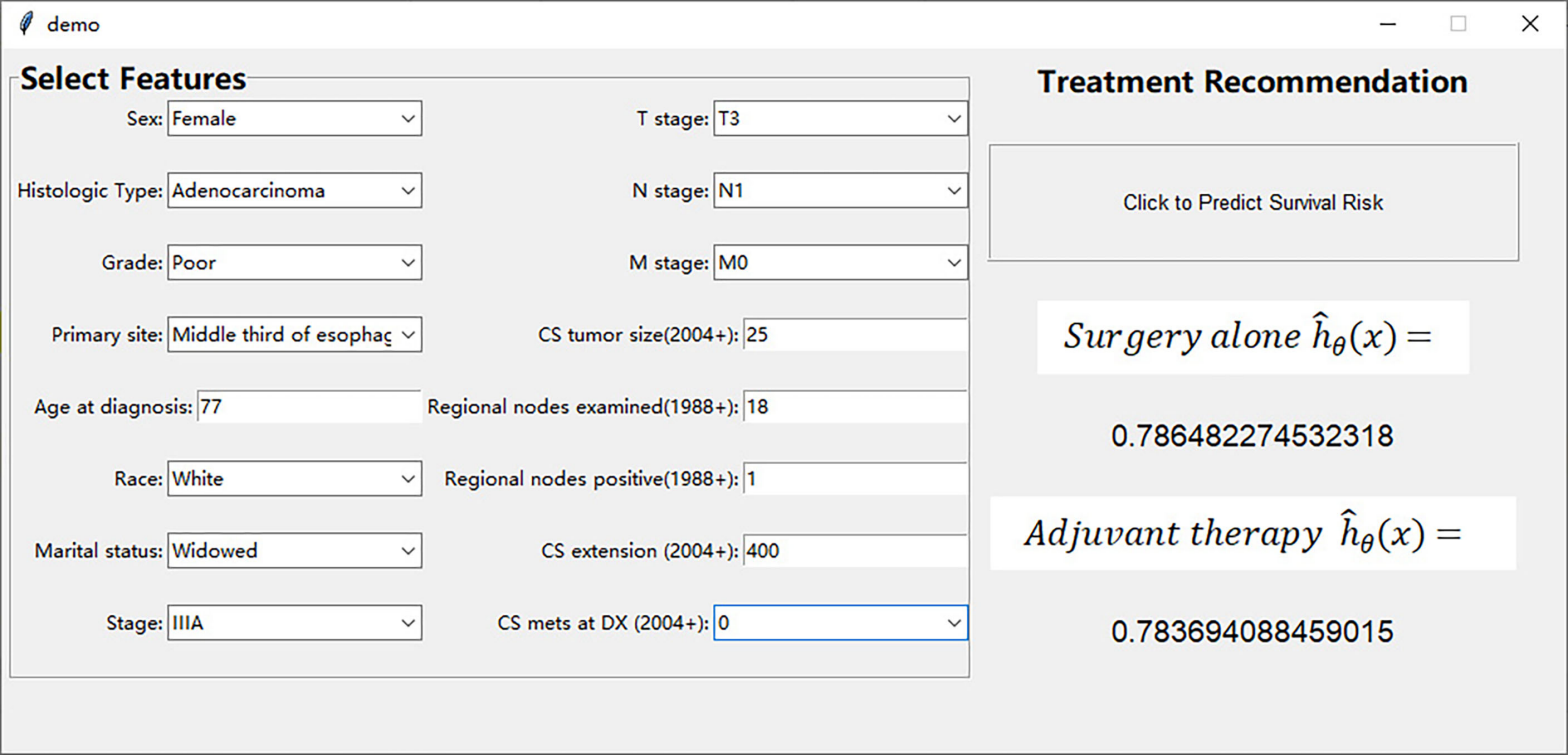
User interface to display the treatment recommendations provided by the DeepSurv model.

We have designed an interactive interface to more intuitively display the treatment recommendations provided by the DeepSurv model. The interface includes user input area on the left and treatment recommendation area on the right (**Figure 5**). Surgeons can input the patient’s prognostic information in the user input area, and click the treatment recommendation button to see the survival risk of different treatment methods in the treatment recommendation area (**Supplement Video**). This interactive interface helps surgeons to choose treatment options with lower survival risk.

## Discussion

This study demonstrated the performance of the DeepSurv model in predicting the prognosis of EC patients, providing treatment recommendations, and found that the performance of the deep learning neural network in predicting ECSS was better than that of the CPH model and TNM staging. Additionally, this study found that patients who followed the treatment plan recommended by the DeepSurv model experienced significantly better ECSS than those who did not.

A series of nomograms have been reported to predict the survival of patients with EC (6–9). Shao et al. (8) established a nomogram to predict the survival of patients with EC undergoing radical resection which included seven variables with the C-index of internal and external validation set as 0.66 and 0.65, respectively. Nomogram is a CPH-based risk prediction model that assumes that the risk of death is a linear combination of its covariates. However, in a real clinical scenario, the assumption that the risk function is linear may be oversimplified. Therefore, a more complex survival model is required to better fit survival data to the nonlinear risk function. Neural networks are widely used in the diagnosis of endoscopic and radiological imaging of EC (19–22), evaluation of the depth of tumor invasion and lymph node metastasis (23, 24), treatment response prediction (25), and in other fields. To date, there have been few studies on the application of deep learning neural networks to survival prediction in patients with EC. Mofidi et al. (26) used artificial neural networks to predict the 1-year and 3-year survival rates of postoperative EC patients, with accuracy rates reaching 88% and 91.5%, respectively, while the accuracy rates of TNM staging were only 71.6% and 74.7%, respectively. Sun et al. developed a survival risk prediction model for EC based on nine blood indices (27). Lin et al. developed a 3D attention autoencoder-based survival prediction network for esophageal cancer using pretreatment CT images (28). Rahman and his colleges demonstrated that the Random Survival Forest model performed better than TNM stage for survival prediction of patients after esophagectomy (29). However, these studies generally have the disadvantages of small sample size and lack of external validation.

DeepSurv, first proposed by Katzman et al., is a multilayer perceptron similar to the Faraggi–Simon network (14). The Faraggi–Simon network is a feed-forward neural network, and its advantage is that it can achieve prognostic prediction without performing feature selection on multiple variables. She et al. found that DeepSurv was superior to the traditional linear model for predicting lung cancer-specific survival (15). In this study, for the first time, the DeepSurv model was used to analyze large-scale EC clinical data, and the model was validated using independent external data, which helped address deficiencies in previous studies. This model includes 19 factors and 96 features, whereas the CPH model constructed with the same data includes only 15 variables. The C statistic of the DeepSurv model was better than that of the CPH model and TNM staging in both the SEER and CHINA test datasets, indicating that the DeepSurv model has better discrimination ability. Meanwhile, the DeepSurv model with a low AIC indicated a better model fit. Baseline clinical characteristics revealed that the training cohort was dominated by white adenocarcinoma patients with stage IV disease, and more than half of the patients did not receive surgical treatment. The external validation cohort included all Asian patients; the pathological type was mostly squamous cell carcinoma and the stage was mostly stage IIB. A better C statistic can still be obtained in the validation data that are completely different from the training set, indicating that the DeepSurv model is superior to the TNM and CPH models in predicting the ECSS.

Currently, there is no consensus regarding the use of adjuvant treatment after radical esophagectomy. Studies have shown that postoperative adjuvant radiotherapy can improve the survival rate of patients with lymph node metastasis (30, 31). For pT2-3N0M0 patients without lymph node metastasis, studies have shown that the use of conformal radiotherapy as postoperative radiotherapy may improve overall survival (OS) and disease-free survival rate (32). A retrospective study reported that postoperative adjuvant chemotherapy could improve the survival rate of patients with esophageal squamous cell carcinoma with lymph node metastasis (33). Postoperative adjuvant chemotherapy is recommended for patients with adenocarcinoma of the esophagus and esophagogastric junction (34). However, no large randomized controlled clinical study has confirmed these conclusions. Deng et al. used the nomogram total score as a reference for postoperative adjuvant treatment in EC (7), and found that for patients with scores between 72 and 227, the 5-year OS rate could be improved by at least 10% through postoperative adjuvant therapy. The advantage of the deep learning model for treatment recommendation is that if the clinical features that affect the prognosis are input into the model, the risk of different treatment plans can be immediately obtained, which is conducive to clinical decision making. In our study, the treatment plan recommended by the deep learning model conferred survival benefits to patients. In subgroup analysis, postoperative adjuvant therapy cannot improve the prognosis of patients with recommendations for surgery alone using the deep learning model. This result is similar to that of previous studies, indicating that very low-risk and very high-risk patients have limited benefits from postoperative adjuvant therapy (7). On the other hand, surgery combined with adjuvant therapy significantly improved ECSS in patients with adjuvant therapy recommendations in the CHINA dataset. Unfortunately, no significant difference in the ECSS was observed between the two treatment opinions in the SEER dataset, which may be related to the lack of samples in the adjuvant therapy recommendation subgroup. Our findings show the potential of the deep learning model as a clinical decision-making tool to help guide patient management.

Clearly, deep learning has advantages in analyzing the nonlinear relationship between clinical features and clinical outcomes; however, there are still shortcomings. First, the function of a deep learning network is similar to a black box, which makes the prediction process difficult to interpret. Second, the deep learning model based on a fully connected neural network is more sensitive to noise, and its feature expression ability and robustness still need to be improved. Although the sample of SEER database is large, however, the SEER database has drawbacks: (1) lack of key pathological features that are closely associated with prognosis, such as marginal status, vessel invasion, resection status (R0/R1/R2); (2) there was no information regarding chemotherapy regimen, drugs, dosage, and toxicities; (3) although there is information on the anatomical target field of radiation, further information on specific radiation type is lacking. These data points are incredibly important for prognosticating survival. Therefore, this model needs to be further improved. In addition, because of the single-center design and insufficient number of cases, the external validation of this model is insufficient, and further studies are needed to verify the advantages of the deep learning network in survival prediction.

## Conclusions

In this study, for the first time, a neural network-based CPH model was used to analyze the relationship between various clinical features and survival outcomes of patients with EC in a real clinical scenario, and satisfactory results were achieved. As a new analytical tool, the DeepSurv model will likely become more widely applied in outcome prediction and treatment recommendations for patients with EC.

## Data availability statement

The data analyzed in this study is subject to the following licenses/restrictions: We obtained permission to access the database after signing and submitting the SEER Research Data Agreement form *via* email. The data that support the findings of this study are available from the SEER database but restrictions apply to the availability of these data, which were used under license for the current study, and so are not publicly available. Requests to access these datasets should be directed to xunyuxu@sina.com.

## Ethic statement

The study was conducted in accordance with the Declaration of Helsinki, and approved by the Ethics Committee of Fujian Provincial Hospital (protocol code k2021-12-042). Patient consent was waived due to the retrospective nature of this study.

## Author contributions

CH: Validation, Writing - Original Draft, Writing - Review & Editing, Visualization. YD: Resources, Writing - Original Draft, Writing - Review & Editing, Visualization. QC: Resources. HC: Methodology, Software. YL: Investigation, Data Curation. JW: Investigation, Data Curation. XX: Conceptualization, Project administration, Funding acquisition. XC: Conceptualization, Methodology, Software, Formal analysis, Supervision. All authors contributed to the article and approved the submitted version.

## Funding

This research was funded by the Special Fund of Fujian Provincial Finance Department [grant number (2021)917] and Natural Science Foundation of Fujian Province [grant number 2022J01412].

## Conflict of interest

The authors declare that the research was conducted in the absence of any commercial or financial relationships that could be construed as a potential conflict of interest.

## Publisher’s note

All claims expressed in this article are solely those of the authors and do not necessarily represent those of their affiliated organizations, or those of the publisher, the editors and the reviewers. Any product that may be evaluated in this article, or claim that may be made by its manufacturer, is not guaranteed or endorsed by the publisher.
