# Supplementary material for: Development and validation of a deep learning model to predict survival of patients with esophageal cancer
Source: Front Oncol. 2022 Aug 10;12:971190. doi: 10.3389/fonc.2022.971190 (PMC9399685; doi:10.3389/fonc.2022.971190)
Supplement: Supplementary file 1 [file DataSheet_1.docx]

Supplementary Material

# Supplementary Tables

**Table S1. All Clinical Features Integrated in the Model**

| Characteristics | Features |  | |  |  |  |  |  |  |  | | |  |  |  |  |  |  |  |  |  |  |  |  |  |  |  |  |  |  |  |
| --- | --- | --- | --- | --- | --- | --- | --- | --- | --- | --- | --- | --- | --- | --- | --- | --- | --- | --- | --- | --- | --- | --- | --- | --- | --- | --- | --- | --- | --- | --- | --- |
| Sex | Female | Male | |  |  |  |  |  |  |  | | |  |  |  |  |  |  |  |  |  |  |  |  |  |  |  |  |  |  |  |
| Histologic Type | Adenocarcinoma | | Squamous cell carcinoma |  |  |  |  |  |  |  | | |  |  |  |  |  |  |  |  |  |  |  |  |  |  |  |  |  |  |  |
| Grade | Well | Moderately | | Poor |  |  |  |  |  |  | | |  |  |  |  |  |  |  |  |  |  |  |  |  |  |  |  |  |  |  |
| Primary site | Upper third of esophagus | Middle third of esophagus | | Lower third of esophagus | Overlapping lesion of esophagus | Cervical esophagus | Abdominal esophagus |  |  |  | | |  |  |  |  |  |  |  |  |  |  |  |  |  |  |  |  |  |  |  |
| Age at diagnosis | 1-101 |  | |  |  |  |  |  |  |  | | |  |  |  |  |  |  |  |  |  |  |  |  |  |  |  |  |  |  |  |
| Race | White | Black | | American Indian/AK Native, Asian/Pacific Islander |  |  |  |  |  |  | | |  |  |  |  |  |  |  |  |  |  |  |  |  |  |  |  |  |  |  |
| Marital status | Widowed | Married | | Single | Divorced | Separated | Unmarried or domestic partner |  |  |  | | |  |  |  |  |  |  |  |  |  |  |  |  |  |  |  |  |  |  |  |
| Stage | IA | IB | | IIA | IIB | IIIA | IIIB | IIIC | IV | | |  |  |  |  |  |  |  |  |  |  |  |  |  |  |  |  |  |  |  |  |
| T stage | T1a | T1b | | T1 NOS | T2 | T3 | T4a | T4b | T4 NOS | | |  |  |  |  |  |  |  |  |  |  |  |  |  |  |  |  |  |  |  |  |
| N stage | N0 | N1 | | N2 | N3 |  |  |  |  | |  | |  |  |  |  |  |  |  |  |  |  |  |  |  |  |  |  |  |  |  |
| M stage | M0 | M1 | |  |  |  |  |  |  | |  | |  |  |  |  |  |  |  |  |  |  |  |  |  |  |  |  |  |  |  |
| Therapy to primary site | Autopsy only | Esophagectomy | | Local tumor destruction |  |  |  |  |  | |  | |  |  |  |  |  |  |  |  |  |  |  |  |  |  |  |  |  |  |  |
| Radiation sequence | No radiation | Radiation prior to surgery | | Radiation after surgery | Radiation before and after surgery | Sequence unknown, but both were given | Surgery both before and after radiation | Intraoperative rad with other rad before/after surgery |  | |  | |  |  |  |  |  |  |  |  |  |  |  |  |  |  |  |  |  |  |  |
| Chemotherapy | Yes | No | |  |  |  |  |  |  | |  | |  |  |  |  |  |  |  |  |  |  |  |  |  |  |  |  |  |  |  |
| CS tumor size(2004+) | 0.1-988mm |  | |  |  |  |  |  |  | |  | |  |  |  |  |  |  |  |  |  |  |  |  |  |  |  |  |  |  |  |
| Regional nodes examined (1988+) | 0-90 |  | |  |  |  |  |  |  | |  | |  |  |  |  |  |  |  |  |  |  |  |  |  |  |  |  |  |  |  |
| Regional nodes positive (1988+) | 0-46 |  | |  |  |  |  |  |  | |  | |  |  |  |  |  |  |  |  |  |  |  |  |  |  |  |  |  |  |  |
| CS extension (2004+)^1^ | 100 | 110 | | 120 | 130 | 160 | 165 | 170 | 200 | | 210 | | 300 | 400 | 450 | 600 | 615 | 650 | 660 | 680 | 710 | 720 | 730 | 745 | 750 | 780 | 800 | 810 | 815 | 820 | 950 |
| CS mets at DX (2004+)^2^ | 00 | 10 | | 11 | 15 | 40 | 50 | 60 |  | |  | |  |  |  |  |  |  |  |  |  |  |  |  |  |  |  |  |  |  |  |

^1^ Details available from: https://web2.facs.org/cstage0205/esophagus/Esophagus_bbb.html

^2^ Details available from: https://web2.facs.org/cstage0205/esophagus/Esophagus_hbg.html

**Table S2. Feature Component Weightings in the DeepSurv Model**

| Features | Weight | Features | Weight | Features | Weight |
| --- | --- | --- | --- | --- | --- |
| Age at diagnosis | -0.252187461 | Derived AJCC Stage Group, 7th (2010-2015)=IA | 0.229776487 | CS extension (2004-2015)=100 | -0.118301801 |
| Race recode=Black | -0.093845271 | Derived AJCC Stage Group, 7th (2010-2015)=IB | 0.429130524 | CS extension (2004-2015)=110 | -0.163202822 |
| Race recode=Other (American Indian/AK Native, Asian/Pacific Islander) | 0.209095716 | Derived AJCC Stage Group, 7th (2010-2015)=IIA | -0.238874123 | CS extension (2004-2015)=120 | -0.042818557 |
| Race recode=White | 0.201880455 | Derived AJCC Stage Group, 7th (2010-2015)=IIB | 0.252297342 | CS extension (2004-2015)=130 | -0.199771687 |
| Marital status=Divorced | 0.7844733 | Derived AJCC Stage Group, 7th (2010-2015)=IIIA | -0.15764375 | CS extension (2004-2015)=160 | 0.291380227 |
| Marital status=Married (including common law) | -0.414804459 | Derived AJCC Stage Group, 7th (2010-2015)=IIIB | -0.549566209 | CS extension (2004-2015)=165 | -0.223242894 |
| Marital status=Separated | -0.282457143 | Derived AJCC Stage Group, 7th (2010-2015)=IIIC | 0.067467168 | CS extension (2004-2015)=170 | 0.041174896 |
| Marital status=Single (never married) | -0.036677688 | Derived AJCC Stage Group, 7th  (2010-2015)=IV | 0.098746583 | CS extension (2004-2015)=200 | 0.172424257 |
| Marital status=Unmarried or Domestic Partner | 0.052207708 | Derived AJCC N, 7th (2010-2015)=N0 | -0.059599623 | CS extension (2004-2015)=210 | -0.155373484 |
| Marital status=Widowed | -0.026857946 | Derived AJCC N, 7th (2010-2015)=N1 | 0.271042466 | CS extension (2004-2015)=300 | 0.01896498 |
| Chemotherapy recode=No/Unknown | 0.222232029 | Derived AJCC N, 7th (2010-2015)=N2 | -0.340484232 | CS extension (2004-2015)=400 | 0.416121721 |
| Chemotherapy recode=Yes | 0.295446634 | Derived AJCC N, 7th (2010-2015)=N3 | 0.249097839 | CS extension (2004-2015)=450 | 0.398490429 |
| Regional nodes examined (1988+)modified | -0.253149688 | Derived AJCC M, 7th (2010-2015)=M0 | 1 | CS extension (2004-2015)=600 | -0.057131886 |
| Regional nodes positive (1988+)modified | -0.357720762 | Derived AJCC M, 7th (2010-2015)=M1 | 0.113892607 | CS extension (2004-2015)=615 | 0.074564308 |
| Sex=Female | 0.292524725 | Derived AJCC T, 7th (2010-2015)=T1a | -0.177147672 | CS extension (2004-2015)=650 | -0.491448343 |
| Sex=Male | 0.415024549 | Derived AJCC T, 7th (2010-2015)=T1b | 0.115860395 | CS extension (2004-2015)=660 | -0.289895117 |
| Histological type=Squamous cell carcinoma | 0.498565525 | Derived AJCC T, 7th (2010-2015)=T1NOS | 0.121728808 | CS extension (2004-2015)=680 | 0.171515107 |
| Histological type=Adenocarcinoma | -0.192998782 | Derived AJCC T, 7th (2010-2015)=T2 | -0.127635837 | CS extension (2004-2015)=710 | 0.156151697 |
| CS tumor size (2004-2015) | 0.14559257 | Derived AJCC T, 7th (2010-2015)=T3 | 0.284216195 | CS extension (2004-2015)=720 | 0.019183539 |
| Therapy to primary site=None | 0.023813874 | Derived AJCC T, 7th (2010-2015)=T4a | 0.071819358 | CS extension (2004-2015)=730 | 0.115353897 |
| Therapy to primary site=Esophagectomy | 0.167601898 | Derived AJCC T, 7th (2010-2015)=T4b | 0.053256061 | CS extension (2004-2015)=740 | -0.652559638 |
| Therapy to primary site=Local tumor destruction or excision | 0.047658034 | Derived AJCC T, 7th (2010-2015)=T4NOS | -0.456854284 | CS extension (2004-2015)=745 | 0.090664878 |
| Grade I | -0.127917215 | Intraoperative rad with other rad before/after surgery | -0.077391051 | CS extension (2004-2015)=750 | -0.000230156 |
| Grade II | -0.151692942 | No radiation and/or cancer-directed surgery | -0.346554548 | CS extension (2004-2015)=780 | 0.03125228 |
| Grade III | -0.146591693 | Radiation after surgery | 0.019661389 | CS extension (2004-2015)=800 | -0.201725811 |
| Primary Site=Cervical esophagus | -0.065683454 | Radiation before and after surgery | -0.505105793 | CS extension (2004-2015)=810 | 0.457207233 |
| Primary Site=Upper third of esophagus | -0.204518139 | Radiation prior to surgery | 0.12235523 | CS extension (2004-2015)=815 | 0.269584805 |
| Primary Site =Middle third of esophagus | -0.578702033 | Sequence unknown, but both were given | -0.055585686 | CS extension (2004-2015)=820 | -0.112792671 |
| Primary Site=Lower third of esophagus | -0.313515812 | Surgery both before and after radiation | 0.767229795 | CS extension (2004-2015)=950 | 0.273699671 |
| Primary Site=Abdominal esophagus | -0.08183711 |  |  | CS mets at DX (2004-2015)=0 | 0.112403966 |
| Primary Site=Overlapping lesion of esophagus | -0.26401642 |  |  | CS mets at DX (2004-2015)=10 | -0.002083116 |
|  |  |  |  | CS mets at DX (2004-2015)=11 | -0.117764689 |
|  |  |  |  | CS mets at DX (2004-2015)=15 | -0.089296728 |
|  |  |  |  | CS mets at DX (2004-2015)=40 | 0.231139705 |
|  |  |  |  | CS mets at DX (2004-2015)=50 | -0.007382277 |
|  |  |  |  | CS mets at DX (2004-2015)=60 | -0.263527244 |

AJCC=American Joint Committee on Cancer

# Supplementary Figure


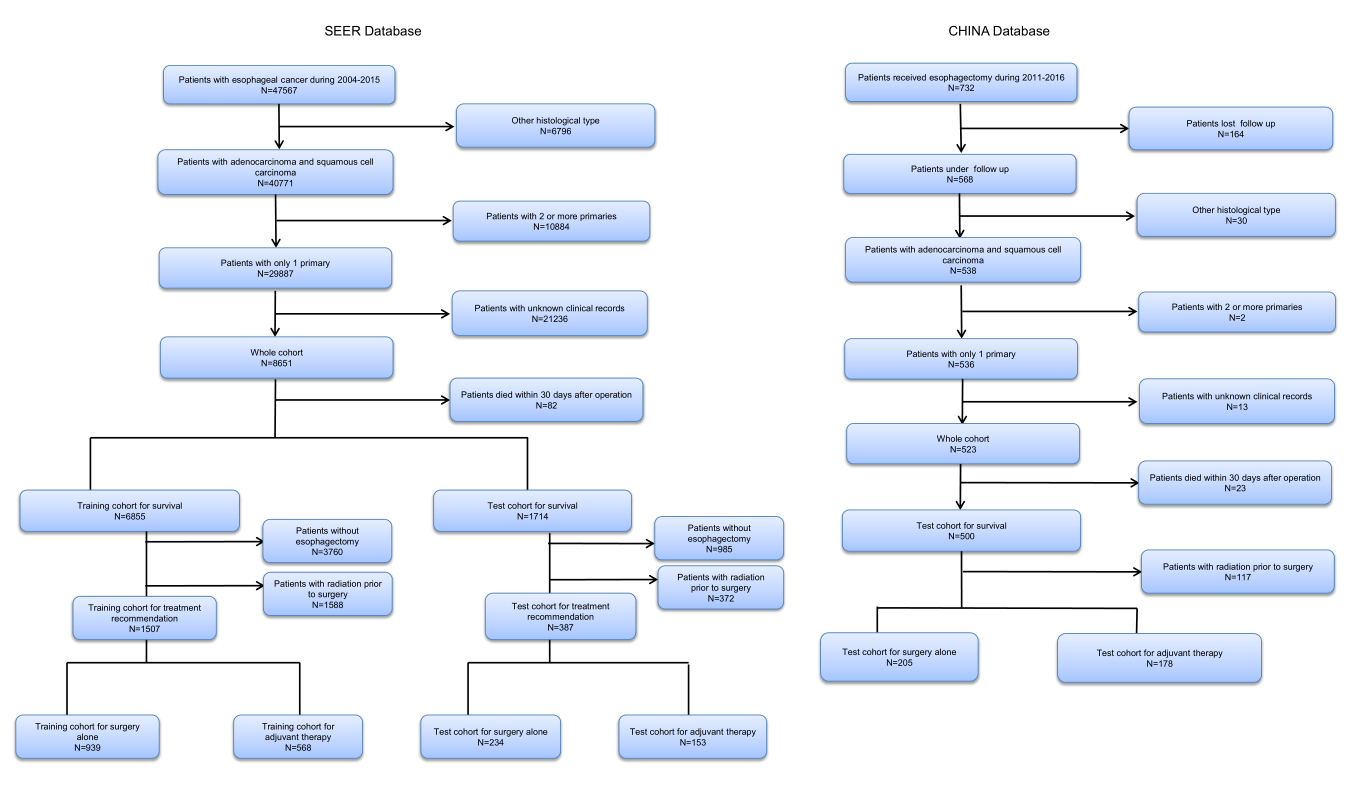


**Figure S1. Flow chart of datasets construction. (A) SEER dataset, (B) CHINA dataset.**

# Supplementary Video

Video S1. User interface to display the treatment recommendations provided by the DeepSurv model.
